# Soaking Up Success: Sponge-Assisted Nanoparticle Transfection

**DOI:** 10.21203/rs.3.rs-8428323/v1

**Published:** 2026-01-30

**Authors:** Yevgeny Brudno, Rukesh Chinthapatla, Hayden Sandt, Israt Jahan Tulip, Vedamudra Kolluru, Hannah Haynes, Savannah Muron, Sharda Pandit, Christopher Moody, Madelyn VanBlunk, Soumen Saha, Ashutosh Chilkoti, Erin Heinzen, Pritha Agarwalla

**Affiliations:** School of Pharmacy at University of North Carolina at Chapel Hill; University of North Carolina at Chapel Hill; University of North Carolina at Chapel Hill; University of North Carolina at Chapel Hill; University of North Carolina at Chapel Hill; University of North Carolina at Chapel Hill; University of North Carolina at Chapel Hill; University of North Carolina at Chapel Hill; University of North Carolina at Chapel Hill; University of North Carolina at Chapel Hill; Duke University; Duke University; University of North Carolina at Chapel Hill; University of North Carolina at Chapel Hill

## Abstract

Current cell transfection systems face efficiency limitations, particularly for challenging cell types. We present a sponge-assisted transfection method enabling efficient mRNA transfection at nanogram-scale doses—a several orders of magnitude-reduction compared to the microgram quantities typically required by lipid-based and membrane-disruption methods. This approach achieves robust, dose-dependent transfection across difficult-to-transfect cell types, establishing broad applicability for both basic research and therapeutic use.

Despite its central role in genetic engineering, cellular therapies, and fundamental biological research, efficient *in vitro* intracellular delivery remains a persistent technical challenge^1,2^. The numerous transfection strategies so-far developed often fail to meet the needs of researchers working with challenging cell types such as immune and stem cells^3,4^. Electroporation, though broadly adopted, causes high cytotoxicity and altered gene expression^5^. Lipid- and polymer-based carriers, while effective in certain immortalized lines, generally require microgram-scale nucleic acid doses and often underperform in recalcitrant primary cells^6^. As a result, delivery challenges continue to limit the accessibility and reproducibility of genetic manipulations across many biological systems.

Here, we report a fundamentally distinct strategy—sponge-assisted transfection—a platform technology based on dissolvable “sponges” that substantially enhances nanoparticle transfection efficiency^7^ (**Fig. S1**). We previously reported that these alginate cryogels enhance viral transduction in primary T cells and enable *in vivo* manufacturing of chimeric antigen receptor (CAR) T cells^8–11^. In this report, we extend their application to nonviral transfection with lipid nanoparticles (LNPs) formulated with the same lipid composition as SPIKEVAX (Moderna)^12^, but encapsulating reporter messenger RNA (mRNA). In contrast to conventional protocols that use microgram-scale mRNA doses per million cells, sponge-assisted transfection achieves similar protein expression using only tens of nanograms, requires no specialized equipment, is scalable, and supports a wide range of vectors and cell types.

We first tested sponge-assisted transfection of Jurkat cells (immortalized human T lymphocytes) using LNPs containing Cy5-labeled, eGFP-encoding mRNA either on dry sponges or empty wells as controls (droplet transfection). We found that sponges dramatically enhanced mRNA uptake (Cy5 signal) and protein expression (eGFP signal), achieving robust signals at just 10ng mRNA and with minimal loss of viability (Fig. 1a–c). In sponges, median fluorescence intensities (MFIs) showed linear dose-dependent behavior up to 100ng mRNA with higher doses inducing saturation effects and decreased viability (Fig. 1d&e). Meanwhile droplet controls achieved signîcantly lower signals at the same dose.

Intriguingly, sponge-assisted transfection exhibited 4-6-fold higher Cy5-to-eGFP MFI (uptake-to-expression) ratios (Fig. 1f&g), suggesting alternative uptake pathways and/or increased endosomal retention. In addition, sponges yielded 18–25-fold higher gene expression at optimal (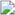
image100 ng) doses (Fig. 1h) and achieved equivalent expression levels with 20–30-fold less mRNA than droplet transfection (Fig. 1i) without noticeably affecting viability. Analysis of individual cellular fluorescence intensities revealed a poor correlation between Cy5 (uptake) and eGFP (expression) signal with both droplet (R^2^ = 0.13 at 1000 ng) and sponge (R^2^ = 0.18), suggesting that endosomal escape - not uptake - remains a bottleneck for mRNA delivery with LNPs (Fig. 1j).

We further benchmarked sponges against electroporation using the commercial Neon NxT system. Despite optimized electroporation parameters (**Fig. S2a-c**), electroporation performed worse than LNPs alone. Electroporated cells yielded broad, heterogeneous fluorescence distributions, while sponge transfection produced tighter, unimodal expression profiles (**Fig. S2d-g**).

Encouraged by the substantial enhancement of LNP uptake, we tested sponges in a longstanding challenge: achieving ratiometric co-expression from separate carriers^13,14^. We evaluated eGFP and mCherry mRNAs at three different molar ratios (3:1, 2:2, and 1:3), either co-encapsulated or separately encapsulated in LNPs (Fig. 2a). As expected, while transfection without sponges could maintain population-level stoichiometry using either co- or separately encapsulated LNPs (Fig. 2b), individual cells often failed to co-express both cargos proportionally (Fig. 2c, **S3**). Co-encapsulating the two mRNAs improved proportional gene expression at the single-cell level, though not always at the intended ratio when benchmarked against single color transfections—likely due to unequal encapsulation efficiencies during nanoparticle formulation (Fig. 2d). Sponge-assisted transfection dramatically increased co-expression, even when using separate LNPs, achieving high single-cell fluorescence correlations comparable to that of droplet transfection using co-encapsulated LNPs (Fig. 2e). This phenomenon may be expression level dependent and emerges due to the sheer efficiency of sponge-assisted transfection (**Fig. S4**). Thus, sponges not only enhance uptake but enable ratiometric co-expression from distinct nanoparticles, opening new possibilities for modular, mix-and-match therapeutic design such as components for CRISPR gene editing.^15,16^

We next systematically evaluated sponge transfection parameters including cell density (with and without scaling LNP dose), droplet volume, incubation time, and hydration state. Sponges performed as expected with a dose-dependent decline in eGFP MFI with increasing cell density with constant LNP dose and maintained eGFP signal when scaling up doses of cells and LNPs together (**Fig. S5a, b**). Transfection efficiencies generally improved with smaller droplet volumes (i.e., higher LNP concentrations). Below ~50 μL, capillary imbibition loads only a small, uneven subset of sponge pores, so cells and LNPs sample fewer microenvironments. This reduced ‘pore averaging’ increased replicate-to-replicate variability and so 50 μL was selected as the optimal droplet volume (**Fig. S5c**). Across 5 to 40 minutes incubation time in the sponge, transfection gains were modest; hence, a 20-minute incubation time was adopted as it consistently achieved near-plateau performance without extending workflow time (**Fig. S5d**). Lastly, we previously observed hydrated sponges had significantly diminished viral transduction enhancement^9^. Here, we also found that hydrated sponges were inferior to dry sponges, however hydrated sponges still yielded markedly higher nanoparticle uptake then controls without sponge, suggesting that hydration state may influence intracellular trafficking pathways (**Fig. S6**). In both cases, sponges reduced proliferation of cells compared to plate controls, though we observed proliferation rates rebound after isolation.

To evaluate the generality of sponge-enhanced cell transduction, we next assessed transfection across 28 cell types: 13 primary cells and 15 immortalized cell lines (Fig. 3). Sponges consistently improved transfection over droplet transfection in primary cells, including T cells, murine HSCs, and equine MSCs. Human NK cells and murine macrophages proved more resistant to transfection requiring higher mRNA doses compared to other cells. Macrophages exhibited right-tailed distributions, reflecting heterogeneous susceptibility to transfection—potentially linked to activation or differentiation states (**Fig S7a**). Among immortalized lines, sponge transfection was generally well-tolerated and efficient. Most lines exceeded 80% eGFP^+^ at just 50 ng mRNA (**Fig. S7b**). More recalcitrant lines including hTERT-RPE1 and ExpiCHO also responded favorably, despite virtually no effectiveness from droplet controls. Overall, no major cytotoxicity was observed, underscoring the general biocompatibility of the system (**Fig. S7c**).

Despite its versatility, sponge-assisted transfection is not without limitations. Sponges were not universally tolerated, with human umbilical vein endothelial cells (HUVECs) exhibiting significant viability loss (~40%) relative to droplet controls (~80%) (**Fig. S7d**). Human induced pluripotent stem cells (iPSCs) and neural progenitor cells (NPCs) also performed poorly with respect to viability. These cells likely suffered from substrate dependence and sensitivity to dissociation. Surviving cells displayed strong fluorescence, indicating successful transfection in a viable subpopulation. Optimization of ECM coatings on the sponges (e.g., using RGD modified alginate) may improve compatibility^17^. While this study focused on mRNA transfection, the performance of this platform with other cargo and carrier combinations warrants further exploration.

Collectively, our work establishes sponge-assisted transfection as a simple, safe, and effective platform for intracellular delivery. The platform’s ability to achieve high gene expression with nanogram-scale doses addresses critical cost and efficacy concerns that currently limit the feasibility of large-scale or repeated transfections. These improvements are especially notable given our use of clinically validated and optimized LNP formulations. The efficiency, versatility, and scalability of sponges make them an attractive alternative to existing methods, particularly for difficult-to-transfect immune cells. This approach presents the potential to accelerate both basic research and translational advances in cell therapy, gene editing, and regenerative medicine.

## Methods

### Fabrication of alginate sponges

Alginate sponges using our ‘Drydux’ formulation was prepared as previously described^7^. In short, 2% w/v ultrapure MVG alginate (4200101, Pronova) was dissolved in sterile filtered DI water and stirred thoroughly. After the alginate was fully dissolved, an equal volume of 0.4% calcium d-gluconate (A11649.36, ThermoFisher) was added, and the mixture was stirred at high speed for 15 minutes. The resulting solution was then aliquoted into 24-well plates (1000 μl per well) and frozen at −20 °C overnight. The following day, the cryogels were transferred to a lyophilizer (7740020, Labconco). Samples were pre-frozen at −20 °C prior to lyophilization to ensure uniform solidification. Before sample loading, the shelf temperature was set to −5 °C, and the chamber was evacuated to below 700 mTorr to allow the system to equilibrate at low temperature. After equilibration, the vacuum was released and the pre-frozen samples were placed onto the lyophilizer shelf. The chamber was then re-evacuated and maintained at approximately 100 mTorr throughout the drying cycle. The shelf temperature was increased in a controlled, stepwise manner according to the following program: hold at +5 °C for 3 h, ramped to +15 °C over 16 h, further increased to +20 °C over an additional 16 h, and subsequently maintained at +20 °C for the remainder of the cycle. To prevent partial melting during chamber stabilization and loading, samples were pre-cooled immediately prior to lyophilization. Although a constant-temperature protocol (20 °C at 100 mTorr) could theoretically be employed, a gradual temperature ramp was adopted to minimize the risk of thermal stress and uneven sublimation associated with rapid warming. Samples were typically removed after a total lyophilization time of 72 h. Prolonged drying beyond this duration occasionally resulted in minor shrinkage of the scaffolds. After the scaffolds were removed, they were stored in a vacuum-sealed bag at 4 °C until needed. Modified sponges were fabrication using the identical process except starting with VLVG (4200501, Pronova) or LVG (4200001, Pronova) alginate or changing percent of calcium d-gluconate.

### Fabrication of lipid nanoparticles

Lipid nanoparticles were formulated similar to Moderna’s mRNA-1273 vaccine formulation. The organic phase consists of molar ratios of SM-102 (Sinopeg):DMG-PEG 2000 (880151P1G, Avanti Polar Lipids):cholesterol (C3045, Sigma Aldrich):DSPC (850365P, Avanti Polar Lipids) at 50:1.5:38.5:10 dissolved in ethanol (04355223, Fisher Scientific). Aqueous phase consists of enhanced green fluorescent protein (eGFP) and/or mCherry (RP-A00041, GenScript) encoding mRNA (RP-A00008, Genscript) dissolved in 10 mM citrate buffer pH 4.0 (J61249.AP, ThermoFisher). The organic and aqueous phases were mixed by rapid pipetting and brief vortex of at a 1:3 (v/v) ratio and 6:1 nitrogen to phosphate ratio. After incubation at room temperature for 15 minutes, the LNP solution was dialyzed for 3 hours at 4 °C in 3.5K MWCO Slide-A-Lyzer^™^ (66333, ThermoScientific) dialysis cassettes against DPBS (14190144, Gibco). The LNPs were stored at 4 °C and used within 1 week of preparation. The encapsulation efficiency was measured using Quant-it^™^ RiboGreen RNA Assay Kit (R11490, Invitrogen) and ranged 60–80% between batches. Dosage of mRNA-LNPs was based on percent of encapsulated mRNA.

### Droplet and sponge-assisted transfection

Droplet transfection was performed by resuspending cells (20 × 10^6^ per mL) in fresh media. Then, 25 μL of this cell suspension was diluted with 25-X μL media and X μL of nanoparticles were added, so the final volume per triplicate is 50 μL. The 50 μL droplet was then pipetted into a well plate and incubated for 20 minutes at 37 °C and 5% CO_2_, before adding additional media for overnight culturing. Suspension cells were incubated in a non-tissue culture treated 24-well plate with 1 mL media, while adherent cells were incubated in a tissue culture treated 12-well plate with 2 mL media. Sponge-assisted transfection was performed similarly to droplet transfection. The 50 μL droplet was added to an alginate sponge in a non-tissue culture treated 24-well plate and incubated for 20 minutes at 37 °C and 5% CO_2_. Additional media (1 mL) was then added and cultured overnight. For transfections using pre-hydrated sponges, the sponges were incubated in media for 24 hours and excess media removed prior to transfection. The hydrated sponges absorbed approximately 0.5 mL of media, and 0.5 mL of additional media was added instead of 1 mL as for a dry sponge.

### Electroporation

Electroporation was perform following the Neon NxT user manual. To optimize for different electroporation parameters, Jurkat cells were electroporated with eGFP mRNA using the Neon NxT electroporation system (Thermo Fisher). Electroporation parameters were systematically optimized by varying voltage, pulse width, and number of pulses across nine distinct protocols. For each condition, 0.5 × 10^6^ Jurkat cells were resuspended in 10 μL of Neon R buffer and electroporated with eGFP mRNA. The electroporation conditions tested were as follows: **Protocol A:** 1410 V, 30 ms, 1 pulse, **Protocol B:** 1700V, 20 ms, 1 pulses, **Protocol C:** 1400 V, 20 ms, 2 pulses, **Protocol D:** 1300 V, 10 ms, 3 pulse, **Protocol E:** 1325 V, 10 ms, 3 pulses, **Protocol F:** 1450 V, 10 ms, 3 pulses, **Protocol G:**1600 V, 10 ms, 3 pulses,. Immediately after electroporation, cells were transferred into pre-warmed RPMI 1640 media supplemented with 10% FBS and cultured at 37°C in 5% CO_2_. Transfection efficiency and viability were assessed 24 hours post-electroporation by flow cytometry. Among the protocols tested, the highest expression with acceptable viability was achieved with **Protocol A.**

### Cell isolation and flow cytometry analysis

At approximately 24 hours, cells were prepared for flow cytometry analysis. Cells incubated in sponges were first isolated by dissolving the sponge. Media in the wells containing sponges was transferred to a flow tube, then 1 mL of 50 mM EDTA in DPBS was added to the well and incubated on an orbital shaker (200 RPM) for 10 minutes at room temperature until the sponge was dissolved. For adherent cells, 50 mM EDTA in 1X TrypLE (A1217701, Gibco) was used to dissolve and dissociate spheroids simultaneously. The solution was then transferred to the flow tube and centrifuged (5 mins, 400 xg) to pellet the cells. The cells were then washed in PBS +Ca/Mg. Similarly, cells incubated in well plates alone were washed in DPBS. TrypLE or trypsin (25200056, Gibco) was used accordingly for adherent cells. After washing, cells were incubated with either Live-or-Dye^™^ 405/452 (32003, Biotium) or Sytox^™^ AADvanced^™^ (S10274, Invitrogen) viability stain according to the manufacturer’s instructions. Cells were finally resuspended in stain buffer (554656, BD Biosciences) and analyzed by flow cytometry (LSRII or LSRFortessa, BD) using FACSDiva^™^ Software. Data was further analyzed using FlowJo version 10.10.10.

### Cell culture

All cells were culture according to American Type Culture Collection’s or manufacturer’s recommended practices unless otherwise noted. Human PBMCs were obtained from buffy coat fractions of healthy donors (Gulf Coast Regional Blood Center) through Lymphoprep density gradient separation (AN1001967, Accurate Chemical and Scientific Corporation). To produce activated human T cells, cryopreserved PBMCs were incubated on 24-well plates (1 × 10^6^per well) coated with 1 μg/ml of agonistic monoclonal antibodies targeting CD3 (130-093-387, Miltenyi Biotec) and CD28 (555725, BD Biosciences) with complete T cell media: 44% HyClone RPMI 1640 media (Cytiva SH20096.01), 44% Click’s media (FUJIFILM Biosciences 9195), 10% FBS (Cytiva SH30396.03), 1% Pen-Step (Gibco 15140122), and 1% Glutamax (Gibco 35050061). At 48 hours, IL-7 (200-07, PeproTech) and IL-15 (200-15, PeproTech) were added to concentrations of 10 ng/ml and 5 ng/ml, respectively. The activated T cells were then used for transfection at 72 hours. To produce activated canine T cells, cryopreserved PBMCs were thawed and cultured on 24-well plates coated with CD3 anti-canine antibodies (clone CA17.6F9, generously gifted by Peter Moore, UC Davis) and CD28 anti-canine antibodies (clone 5B8-APC, eBioscience, Invitrogen) at 2 μg per well. The cells were further cultured in complete canine T cell media (Advanced RPMI 1640 (Gibco 12633012) with 10% FBS (Corning 35076CV), 1% Pen-Step (Gibco 15140122), 1% HEPES pH7.3 (Thermo Scientific Chemicals J16924.K2), and 1% Glutamax (Gibco 35050061)) with human IL-2 (500 U ml^−1^) (200-02, PeproTech) for 72 hours before transfection. Human NK cells were isolated from cryopreserved PBMCs following manufacturer’s protocols for NK Cell Isolation Kit, human (130-092-657, Miltenyi Biotec) and expanded using NK MACS media (130-114-429, Miltenyi Biotec), and transfected by day 14. Normal human dermal fibroblasts (CC-2511), human preadipocytes (PT-5020, Lonza), and HUVECs (CC-2517, Lonza) were purchased from Lonza and cultured according to manufacturer’s recommended practices.

*Macaca nemestrina* non-human primate (NHP) PBMCs were obtained from blood samples collected from donors (Washington National Primate Research Center) through Percoll density gradient separation. In short, the cells were initially spun down (1400xg for 15 minutes) and excess plasma removed, diluted in 1 X HBSS with EDTA (100 mg/mL), added to the top of a warmed Percoll solution and spun again (1000 g for 30 minutes with breaks off). Then, band of white blood cells are collected and washed. Cells were further cultured using complete NHP T cells media: Lonza^™^ BioWhittaker^™^ X-VIVO^™^ 15 Hematopoietic Serum-Free Culture Media with Gentamicin, without Phenol Red (Lonza 04418Q) supplemented with 10% FBS (Cytiva SH30396.03), 1% Pen-Step (Gibco 15140122), and 50 μM betamercaptoethanol (Gibco 21985023). To produce activated NHP T cells, cryopreserved PBMCs were incubated on 24-well plates (1 x 10^6^) coated with 1 ug/mL of agonistic monoclonal human antibodies targeting CD3 (Clone SP34-2) and CD28 (Clone CD28.2). Human antibodies with specified clones were cross-reactive with NHP T cells. At 48 hours, human IL-7 and IL-15 were added to concentrations of 10 ng/mL and 5 ng/mL, respectively. The activated T cells were then used for transfection at 72 hours.

iPSC maintenance and culture - Doxycycline inducible Ngn2-expressing human induced pluripotent stem cells (Alstem, iP11NA) were cultured at 37°C with 5% CO_2_ in mTeSR+ medium (Stem Cell Technologies Catalog #100-0276). The cells were fed every 24 hours and passaged every 4-5 days with EZ-Lift (Sigma Aldrich SCM139) according to the manufacturer’s protocol. Cells were kept on Matrigel (Corning 354230) coated plates, diluted in DMEM/F12 (Fisher Scientific 11-330-032) and prepared according to the Alstem Product Specification protocol. iPSCs for this experiment were maintained until an approximate 60% confluency. The cells were then washed twice with 1x PBS (Fisher Scientific 10-010-049), and Accutase (Thermo Scientific A1110501) diluted 1:1 with PBS was added to the cells. After 1 minute at room temperature, the Accutase was removed, and the cells were washed twice with DMEM/F12. mTeSR+ was then added to the wells and the cells were scraped to detach them and transferred to a conical tube to be spun down at 300xg for 5 minutes. The supernatant was removed, and the iPSCs were then resuspended to a single cell state and proceeded to be transfected.

NGN2 neuronal differentiation - On Day 0, any areas of differentiation were removed from the iPSCs (Alstem, iP11NA), and they were washed twice with 1x PBS. Accutase, diluted 1:1 in PBS, was added to the cells and kept at room temperature for 1 minute. It was removed and the cells were gently washed twice with DMEM/F12. mTeSR+ was added to the wells and cells were detached using a cell scraper. They were then transferred to a conical tube, and the wells were rinsed twice with mTeSR+, which was collected in the tube as well. The cells were centrifuged at 300xg for 5 minutes at room temperature, then the supernatant was aspirated, and the iPSC pellet was resuspended in mTeSR+ containing 5 μM of ROCK inhibitor Y-27632 (Hello Bio, HB2297). Cells were counted and plated into a Matrigel coated plate at 7.5 x 10^4^ cells per well of a 6 well plate in small aggregates of 3-5 cells per clump. The iPSCs were distributed across the plate and cultured as normal overnight. On Day 1, old media was replaced by mTeSR+. On Day 2, old media was replaced with N2 media, consisting of: 100x N-2 supplement (Thermo Scientific, 17502-048), 100x GlutaMAX supplement (Thermo Scientific, 35050061), 100x Non Essential Amino Acids (Fisher Scientific, 11-140-050), and 100x Penicillin/Streptomycin (Fisher Scientific, 15-140-122), in DMEM/F12. On day 2, 2 μg/mL Doxycycline (Sigma-Aldrich, D9891-1G) was added to the media as well. On Day 3 and Day 5, the old media was replaced with N2 media containing 2 μg/mL Doxycycline. On Day 6, the cells are now considered immature neurons. The old media was removed from the cells, and they were washed once with 1x PBS. Accutase diluted 1:1 with PBS was added to the cells and left to stand for 2-4 minutes at room temperature. The cells were then carefully picked up and added to a conical of N2 media, centrifuged at 200xg for 5 minutes, and the supernatant was removed. The neurons were carefully resuspended to a single cells state and proceeded to be transfected. Protocols adapted from Product Specification Sheet for iP11NA from Alstem.

## Supplementary Material

This is a list of supplementary files associated with this preprint. Click to download.

• Figures202512221.jpg

• SupplementalFigure1.png

• SupplementalFigure2.png

• Figures202512225.jpg

• SupplementalFigure3.png

• SupplementalFigure4.png

• Figures202512227.jpg

• SupplementalFigure5.png

• Figures202512228.jpg

• SupplementalFigure6.png

• SupplementalFigure7.png

## Figures and Tables

**Figure 1:**
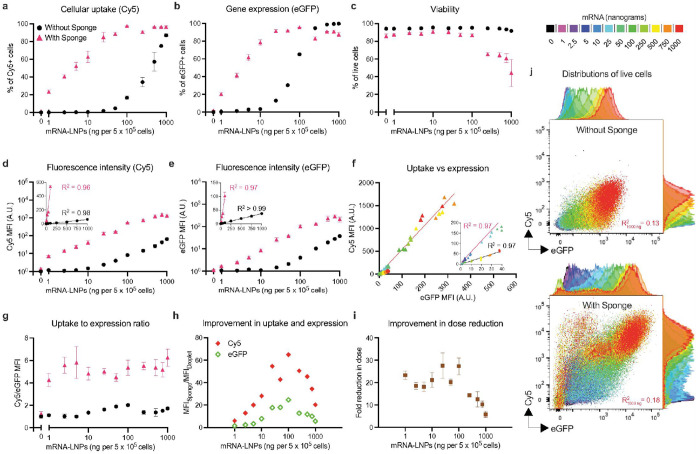
Sponges maximize intracellular transfection of lipid nanoparticles. Transfection of Jurkat cells (n=3 per group) using Cy5 labeled eGFP mRNA-LNPs with sponge-assisted transfection (pink triangles) or droplet transfection (black circles).

**Figure 2:**
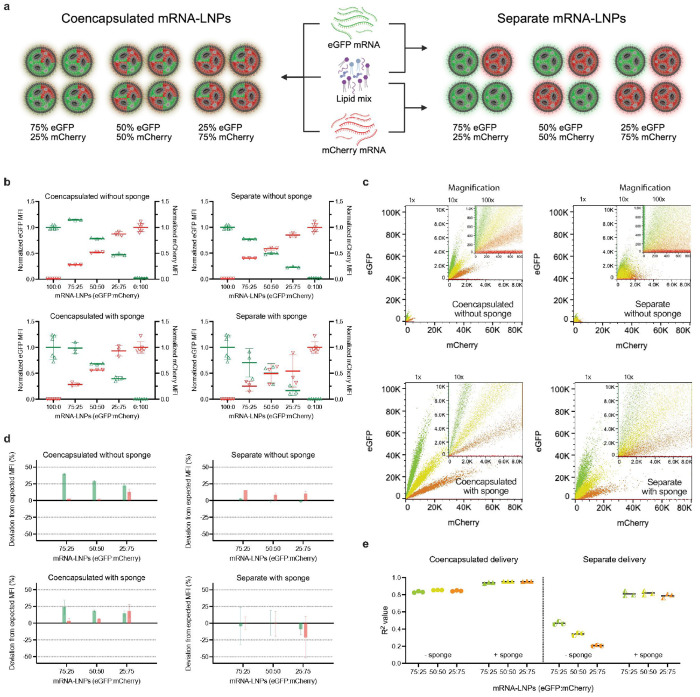
Sponges enable ratiometric transfection of multiple cargos. **a**, Schematic of lipid nanoparticles (LNPs) delivered containing eGFP and/or mCherry fluorescent reporter messenger RNA (mRNA) either together (left) or separately (right). Total dosage of LNPs delivered in each group is 100 nanograms of eGFP to mCherry mRNA at ratios of 100:0, 75:25, 50:50, 25:75, and 0:100 per 250,000 Jurkat cells. **b**, Normalized median fluorescence intensities (MFI) of transfected cells, showing eGFP (green triangles) and mCherry (red nablas). **c**, Representative eGFP by mCherry dot plots. Green, lime, yellow, orange, and red represent ratios of 100:0, 75:25, 50:50, 25:75, and 0:100, respectively. Inserts are magnified to show lower intensities by 10x or 100x. **d**, Relative eGFP (green) and mCherry (red) translation, computed by comparing each co-transfection ratio to the expected MFI obtained by linearly scaling the single-color 100:0 (eGFP) or 0:100 (mCherry) controls at the same total dose, and reporting the percent deviation for conditions with and without sponges. **e,** Coefficient of determination (R^2^) for linear regression lines calculated from dot plots for each sample.

**Figure 3:**
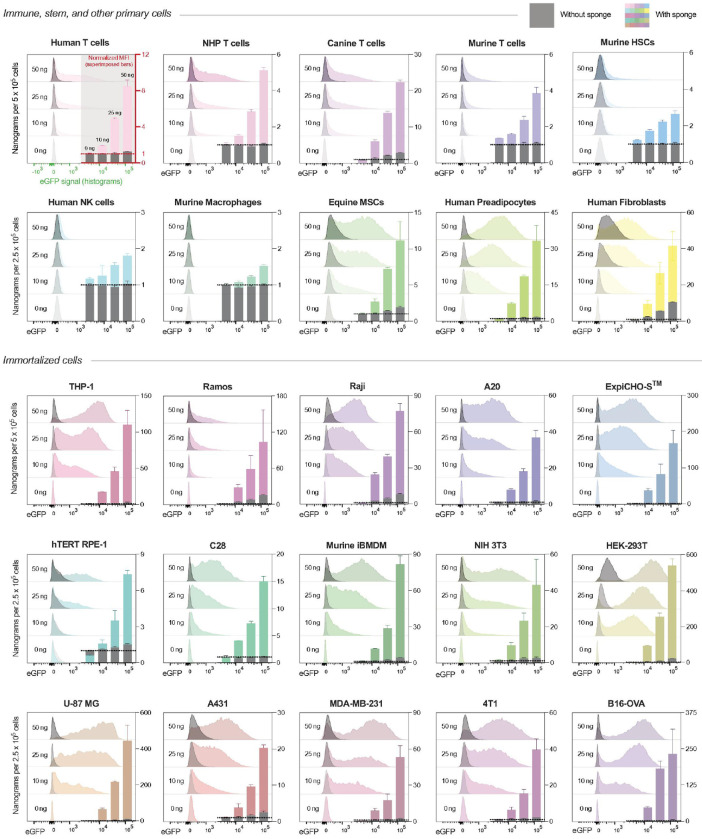
Sponges enable efficient transfection of primary and immortalized cell lines using nanograms of mRNA. Primary (top panels) and immortalized (bottom panels) cells were transfected with eGFP reporter mRNA encapsulated in SM-102 LNPs. Cells were treated with 10, 25, or 50 ng of mRNA to transfect either 500,000 suspension cells (rows 1 and 3) or 250,000 adherent cells (rows 2, 4, and 5). Negative controls (0 ng) were not treated with mRNA-LNPs. Histograms display eGFP expression, comparing droplet (black/grey) versus sponge-assisted transfection (colored). Histograms are overlaid and grouped by dose. Bar graphs (insert) show median fluorescence intensity (MFI) normalized to untreated cells.

## References

[1] Stewart MP (2016) In vitro and ex vivo strategies for intracellular delivery. Nature 538:183–192 27734871 10.1038/nature19764

[2] Morshedi Rad D (2021) A comprehensive review on intracellular delivery. Adv Mater 33:e2005363 33594744 10.1002/adma.202005363

[3] Kumar ARK, Shou Y, Chan B, L, K., Tay A (2021) Materials for improving immune cell transfection. Adv Mater 33:e2007421 33860598 10.1002/adma.202007421

[4] Wells-Holland C, Elfick A (2023) Transfection reflections: fit-for-purpose delivery of nucleic acids. Nat Rev Mol Cell Biol 24:771–772 37344633 10.1038/s41580-023-00627-6

[5] Batista Napotnik T, Polajžer T, Miklavčič D (2021) Cell death due to electroporation - A review. Bioelectrochemistry 141:107871 34147013 10.1016/j.bioelechem.2021.107871

[6] Kucharski M, Mrowiec P, Ocłoń E (2021) Current standards and pitfalls associated with the transfection of primary fibroblast cells. Biotechnol Prog 37:e3152 33774920 10.1002/btpr.3152

[7] VanBlunk M, Agarwalla P, Pandit S, Brudno Y (2022) Fabrication and use of dry macroporous alginate scaffolds for viral transduction of T cells. J Vis Exp. 10.3791/64036 36156536

[8] Lenti -X transduction sponges. https://www.takarabio.com/products/gene-function/viral-transduction/lentivirus/transduction-sponges.

[9] Agarwalla P (2020) Scaffold-mediated static transduction of T cells for CAR-T cell therapy. Adv Healthc Mater 9:e2000275 32592454 10.1002/adhm.202000275 PMC7518635

[10] Agarwalla P (2022) Bioinstructive implantable scaffolds for rapid in vivo manufacture and release of CAR-T cells. Nat Biotechnol 40:1250–1258 35332339 10.1038/s41587-022-01245-x PMC9376243

[11] Pandit S (2024) Implantable CAR T cell factories enhance solid tumor treatment. Biomaterials 308:122580 38640784 10.1016/j.biomaterials.2024.122580 PMC11125516

[12] Hou X, Zaks T, Langer R, Dong Y (2021) Lipid nanoparticles for mRNA delivery. Nat Rev Mater 6:1078–1094 34394960 10.1038/s41578-021-00358-0 PMC8353930

[13] Zhang H (2022) Together is better: mRNA co-encapsulation in lipoplexes is required to obtain ratiometric co-delivery and protein expression on the single cell level. Adv Sci (Weinh) 9:e2102072 34913603 10.1002/advs.202102072 PMC8811815

[14] Moradian H, Lendlein A, Gossen M (2020) Strategies for simultaneous and successive delivery of RNA. J Mol Med 98:1767–1779 33146744 10.1007/s00109-020-01956-1 PMC7679312

[15] Kazemian P (2022) Lipid-nanoparticle-based delivery of CRISPR/Cas9 genome-editing components. Mol Pharm 19:1669–1686 35594500 10.1021/acs.molpharmaceut.1c00916 PMC9176214

[16] Wu F (2025) Lipid nanoparticles for delivery of CRISPR gene editing components. Small Methods e2401632 40434188 10.1002/smtd.202401632 PMC12825352

[17] Yu J (2010) The use of human mesenchymal stem cells encapsulated in RGD modified alginate microspheres in the repair of myocardial infarction in the rat. Biomaterials 31:7012–7020 20566215 10.1016/j.biomaterials.2010.05.078

